# Detection of precancerous lesions in the cervix and HPV infection in women in the region of Maniapure, Bolivar State

**DOI:** 10.3332/ecancer.2018.884

**Published:** 2018-12-03

**Authors:** Andrés Fuenmayor, Carlos Fernández, Verónica Pérez, José Coronado, Maira Ávila, Andreína Fernandes, Jairo Fuenmayor

**Affiliations:** 1 Luis Razetti School, Faculty of Medicine, Central University of Venezuela, Caracas 1050, Venezuela; 2 Molecular Genetics Laboratory, Institute of Oncology and Haematology, Ministry of Health, Caracas 1050, Venezuela; 3 Faculty of Medicine, Central University of Venezuela, Caracas 1050, Venezuela

**Keywords:** precancerous lesions, HPV, Schiller test, visual inspection, cervix

## Abstract

Human papillomavirus (HPV) is the causative agent of cervical cancer (CC), the second most common cause of cancer deaths in Venezuela. Early detection and prompt treatment of precancerous lesions prevent up to 80% of CC cases. In Venezuela, difficult access to CC screening means that the disease is detected at advanced stages, especially in more vulnerable indigenous populations. The aim of the study was to detect precancerous cervical lesions and HPV infection in 60 women who attended the gynaecology service at the Maniapure Outpatient Clinic in Bolivar State, Venezuela. The study was carried out to detect precancerous cervical lesions using visual inspection with acetic acid (VIA), the Schiller test and conventional cytology (Pap testing). HPV detection and typing were carried out using the polymerase chain reaction. 58.3% of the women in the study belonged to the Eñepa indigenous community and 41.7% were white Creole women. The Schiller test showed irregularities in the staining of the exocervical epithelium in 8.33% of the patients, suggesting HPV infection. VIA was positive for 10.0% of the women. In the cytopathology report, 81.67% tested negative for intraepithelial lesions. The overall frequency of HPV detection was 35.0%. HPV infection was detected in 45.71% of the Eñepa women and 20.0% of the Creole women. 71.43% of the women had a high-risk single HPV infection. The percentage of viral infection was lower in the Creole patients than in the indigenous population; therefore, CC screening programmes in the latter population need to be improved.

## Introduction

Cervical cancer (CC) is the second most frequent cause of malignancy and death in women around the world, preceded only by breast cancer [1, 2]. Of the 500,000 new cases diagnosed each year, an estimated 80% originate in developing countries and about 250,000 women die annually because of complications of this disease [3]. In 2012, the standardized incidence and mortality rates for CC per 100,000 women in Venezuela were 27.35 and 11.80, respectively, making it the second leading cause of cancer deaths among Venezuelan women [4].

The causative agent of CC is human papillomavirus (HPV), the most common sexually transmitted viral infection worldwide [5]. It is estimated that more than 290 million women around the world are infected with this virus. Estimates of HPV prevalence vary from 14% to more than 90%, with developing countries being the hardest hit [3, 5].

HPV is a small DNA virus of the *Papillomaviridae* family, which has specific tropism for keratinocytes [6]. It has a genome of approximately 8,000 base pairs (bp), comprising eight early open reading frames (ORFs), two late ORFs and a non-coding control region. The main high-risk oncoproteins are E6 and E7, which inactivate the p53 tumour-suppressor protein and the retinoblastoma protein, respectively, thereby increasing the rate of cell proliferation, which leads to the development of malignancies [7].

In general, many of the genital infections caused by this virus are associated with benign tumours, which usually disappear spontaneously after a period of months or years, and are considered transient viral infections that can remain undetectable in about 90% of women. Only a small number of cases of benign lesions may progress to malignancy and cancer [8, 9]. Persistent infection with high-risk HPV, including genotypes 16, 18, 31, 33, 35, 39, 51, 52, 53, 56, 59, 68 and 73, leads to the development of cervical lesions and CC in approximately 5% of cases [3, 9–12].

Specifically, standardised incidence and CC mortality rates of 28.46 and 12.90 per 100,000 women, respectively, were recorded in Bolivar State, making CC the most frequent cause of cancer deaths in this state [4]. Bolivar, one of the 24 federal divisions of Venezuela, is located in the south-east of the country and is home to many native ethnic groups, who represent 4% of the population. The region of Maniapure, located in the municipality of Cedeño, covers an area of 1,500 square kilometres between Caicara del Orinoco and Pijiguaos in the north-west of the state [13].

It is a low-income region that is difficult to access by land, and its geographical features and the socio-cultural characteristics of the indigenous populations hinder the implementation of health programmes and programmes to screen for and diagnose preinvasive cervical lesions. Maniapure is sparsely populated, with 42 villages or communities, 34 being Creole and 8 being populated by the Panare (also known as the Eñepa), an indigenous Venezuelan ethnic group [13].

Culturally, the Eñepa are one of the strongest indigenous peoples in the state because of their characteristic resistance to acculturation. They are a very closed, monogamous ethnic group, with deep-rooted beliefs and customs: sexually mature girls usually marry at an early age, between 13 and 15 years, and the man is always the head of the household and sometimes speaks Spanish, which is mostly restricted for women [13].

In developed countries, early diagnosis and early treatment of precancerous lesions prevent up to 80% of CC cases. However, in developing countries like Venezuela, difficult access to CC screening means that the disease is detected at more advanced stages [3].

Despite many health organizations aiming to improve the health of indigenous populations, the epidemiological and health profiles of indigenous women are unknown, partly because of a lack of research and the poor information systems that manage the morbidity and mortality of these groups [14].

In South America, some studies have reported the prevalence of HPV and cervical intraepithelial neoplasia in indigenous women in the jungle, while others have assessed indigenous groups in more urbanized regions of the continent. An HPV prevalence of between 14% and 60% in asymptomatic women has been reported in populations that interact with urbanized societies [14].

Given that CC is a national public health issue and that the risk of this disease in indigenous populations, particularly in Maniapure, is understudied, it was decided to carry out this research in indigenous as well as Creole communities, in order to detect precancerous lesions and HPV in the cervix in the female population of Maniapure.

## Methods

This study was part of a descriptive, cross-sectional, observational investigation.

### Population and sample

The population consisted of all the patients who attended the gynaecological service at La Milagrosa Type II Outpatient Clinic in Maniapure, in the month of August 2016, during the third edition of the Multidisciplinary University Camp for Services and Research (CUMIS). From this population, a non-probabilistic, non-intentional sample of 60 patients was taken.

## Inclusion/exclusion criteria

**Inclusion criteria:** sexually active women, regardless of age.

**Exclusion criteria:** sexually mature but non-sexually active women; menstruating women; women who, 48 hours prior to the exam, have engaged in sexual intercourse or used vaginal douches, tampons, soaps, vaginal creams or vaginal medications; hysterectomised women.

This study was approved by the National Bioethics Centre (CENABI). For sampling, the patients were asked to give their prior and written informed consent. They were also given a survey to ascertain their understanding of sexually transmitted infections.

Each patient was screened for precancerous cervical lesions using the tests proposed by WHO: visual inspection with acetic acid (VIA), conventional cytology (Pap testing) and molecular detection of HPV.

### Procedure

The purpose of the study was verbally explained to each patient who met the aforementioned criteria, and they were asked to give written informed consent to take part. The protocol proposed by WHO in 2013 [15] for investigating precancerous cervical lesions, in order to provide primary care was then applied. The protocol includes conventional cytology (Pap testing), VIA and the Schiller test. HPV detection by polymerase chain reaction (PCR) and HPV typing by multiplex PCR were also added.

#### Sample collection for cervical cytology (PAP smear)

The patients were physically examined by gynaecologists and then put in the lithotomy position on a clean sheet to examine the external genitalia. A sterile Graves speculum was inserted into the vagina in order to take a sample of the stratified squamous epithelium from the exocervix by passing an Ayre spatula over the external cervix os. A sterile swab was then inserted into the cervical canal and rotated 360° to take a sample of the endocervical columnar epithelium. The samples were smeared on a slide and fixed with 96% alcohol, then placed in slide-holders that were identified beforehand with the patient’s data. They were subsequently processed and dyed using Pap staining at the Caracas Medical Centre. The results were reported according to the 2014 Bethesda System [16]. The swabs were then collected for HPV detection and typing before the application of acetic acid, Lugol’s iodine or treatment.

#### Collection of the cervical swab sample

The swabs of the transformation zone were taken by inserting the swab past the squamocolumnar junction (SCJ), to collect cervical cells scraped from the endocervical canal up to the exocervix. The swabs were placed in sterile tubes with a transport medium and taken to the Molecular Genetics Laboratory at the Institute of Oncology and Haematology for processing.

#### VIA and the Schiller test

VIA was conducted by applying a 3%–5% acetic acid solution to the cervix. The test was considered positive if distinct, well-defined, dense acetowhite areas were seen and negative if there were no acetowhite lesions.

The Schiller test was carried out by examining the cervix and vagina by using a Graves speculum and applying Lugol’s iodine to the exocervical epithelium. A positive outcome was indicated by well-defined, bright yellow areas without iodine uptake and which abutted the SCJ or were close to external cervix os if the SCJ was not visible.

#### HPV detection by PCR

Genomic material was isolated using the PureLink Genomic DNA Kit (Invitrogen), following the instruction manual.

HPV was identified by conventional PCR using the *MY09/MY11* consensus primers, which amplify a highly conserved 450-bp region of the *L1* gene that encodes a viral capsid protein and allow a broad spectrum of HPV genotypes to be amplified. The *PC04/GH20* primers were included as an internal control for the reaction, to amplify a 268-bp band of the β*-globin* gene.

A 50 μl PCR mixture was prepared, consisting of 1 μg of DNA incubated with: 6.25 μl of 10X buffer, 0.4 μl of dNTPs (100 mM), 0.2 μl of each of the MY09 and MY11 primers, 4 μl of MgCl^2^ (50 mM), 1.2 μl of the β*-globin* primers, 0.5 μl of Taq DNA polymerase (5 U/μl), and nuclease-free distilled water to complete the volume.

The amplification reaction was carried out in a Mastercycler ep thermal cycler (Eppendorf), under the following conditions: initial denaturation at 94°C for 4 minutes, followed by 40 cycles of denaturation at 94°C for 15 seconds; hybridization at 55°C for 30 seconds; extension at 72°C for 45 seconds; and, lastly, final extension at 72°C for 7 minutes. In all the amplification reactions, purified DNA from HPV18-infected HeLa cells was included as a positive control, and nuclease-free distilled water was used as a negative control. The amplification products were viewed using electrophoresis on 2% agarose gels stained with SYBR Safe (Invitrogen). The photographic record was made with a ChemiDoc XRS imaging system (BioRad).

A sample was considered positive if the 450-bp band corresponding to the HPV L1 region and the 268-bp band corresponding to the β*-globin* were amplified, and negative if only the 268-bp band was amplified.

#### HPV typing by multiplex PCR

Viral typing of the positive samples was performed through multiplex PCR with the Seeplex HPV4 ACE Genotyping kit (Seegene, Inc.), which identifies two low-risk genotypes (such as 6 and 11), two high-risk genotypes (such as 16 and 18) and a band of high-risk genotypes (HRC) that includes types 26, 31, 33, 35, 39, 45, 51, 52, 53, 56, 58, 59, 66, 68, 73 and 82. The kit also has a 1000-bp internal control. The amplification products were viewed using electrophoresis on 2% agarose gels stained with SYBR Safe (Invitrogen). The photographic record was made with a ChemiDoc XRS imaging system (BioRad). Subsequently, the results were analysed.

### Statistical analyses

For continuous variables, averages and standard deviations were used, and frequency analysis and contingency tables were used for discrete variables. The Fisher test and analysis of variance were performed using the statistical program R version 3.2.3. A value of *p* < 0.05 was obtained, with an odds ratio of 3.36 and a 95% confidence interval.

## Results

Of the 60 women who participated in the study, 100% accepted the conditions of informed consent for the detection of precancerous lesions of the cervix, through VIA, the Schiller test, conventional cytology (Pap test) and molecular detection of HPV infection, 58.3% (35/60) belonged to the indigenous community of Eñepa and 41.7% (25/60) were Creole women from the region of Maniapure, Bolivar state.

The sociodemographic characteristics of each population evaluated are shown in Table 1. The Creole population had a slightly higher average age than the indigenous population, with no statistically significant differences. The majority of women, both Creole and indigenous, were married and responsible for the housework. Statistically significant differences were found between both populations regarding the use of oral contraceptives, knowledge of STIs and whether they know how to avoid the spread of STIs.

**Figure 1.**
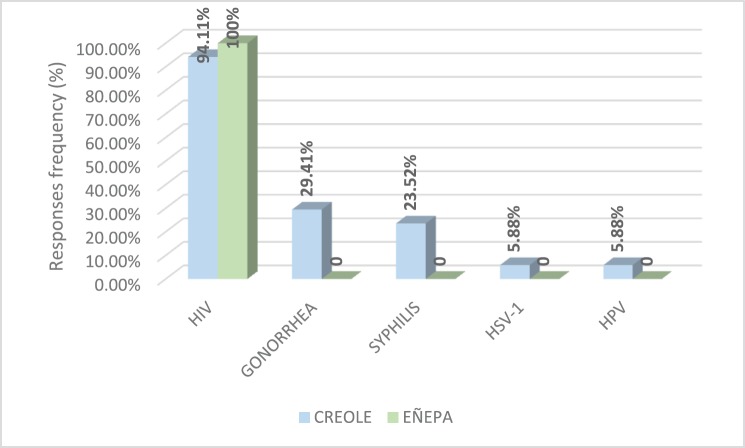
Sexually transmitted infections known to the population of women, both Creole and indigenous.

Based on the answers given by study participants to a survey, Figure 1 shows the STIs they had knowledge of. According to this, only 5.88% (1/17) of the Creole patients said they knew HPV as an STI. Infection by the human immunodeficiency virus was the most common in both populations.

Upon analyzing the results of the Schiller test, it was observed that of the 60 women participating in the study, only 8.33% (5/60) showed abnormalities in the stain of the exocervical epithelium, with areas of low uptake being compatible with an abnormality, suggestive of HPV infection. During the visual examination with acetic acid, 10.0% (6/60) of the women evaluated were found to be positive for this test. Of the total population, 81.67% (49/60) were negative for intraepithelial lesions on the cytopathological report.

Table 2 shows the distribution of patients according to the population to which they belonged, based on the results of the Schiller test, VIA, cytology and HPV detection. In the group of Creole women was a single case of low-grade intraepithelial lesion (LSIL), while in the Eñepa population there were three cases of LSIL, one case of high-grade intraepithelial lesion (HSIL) and one report of ASCUS. HPV infection was detected in 35.00% (21/60) of all of the women studied. Table 2 shows the results of HPV detection in cervical swabs taken from the study population. The highest frequency was reported in the Eñepa community, demonstrating a statistically significant difference.

In the indigenous population, for those that were positive for HPV, the average age was 23.31 ± 10.10 years, compared with the negative HPV group, which had an average age of 35.53 ± 16.72 years (*p* = 0.015). In Creole population, the group of women that tested positive for HPV had an average age of 36.20 ± 11.39 years, while those negative for HPV had an average age of 32.20 ± 11.42 years (*p* = 0.490).

Regarding the evaluation of the HPV genotypes, it was observed that of the 21 women infected with HPV, 71.43% (15/21) had an infection with high-risk genotypes, the most common being HRC, which includes the genotypes 26, 31, 33, 35, 39, 45, 51, 52, 53, 56, 58, 59, 66, 68, 73 and 82 with 60.0% (9/15), followed by type 18 with 33.33% (5/15) and type 16 with 6.67% (1/15). In 28.57% (6/21) of cases, the genotype could not be identified with the method used.

**Figure 2.**
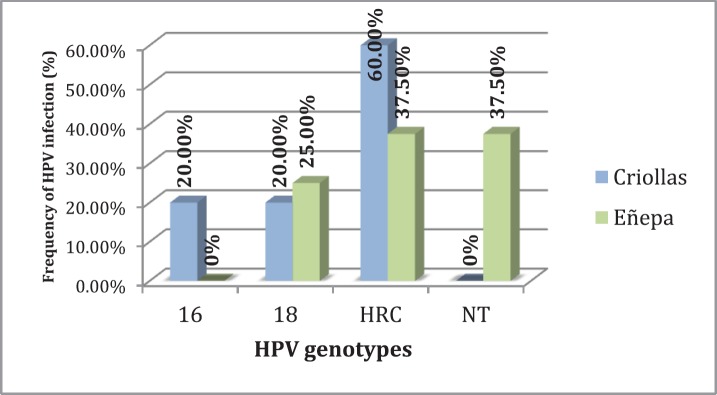
Frequency of HPV genotypes present in the study populations. HRC = high-risk genotypes; NT = not typable.

Specifically, in the indigenous community, infection with high-risk genotypes was detected in 28.57% (10/35) of the study population, while in the Creole community it was detected at 20.0% (5/25). Figure 2 shows the distribution of genotypes found, both in the indigenous community and in the Creole community.

Linking the results of cervical cytology, the Schiller test and VIA, with the detection of HPV infection using PCR, it was noted that only one patient (2.85%) belonging to an indigenous community presented with an HSIL, with a Schiller test with low update compatible with HPV infection and which detected the HRC band. Furthermore, three patients had LSIL, of which two were negative for other tests and one presented with of non-typeable HPV infection, with positive VIA and Schiller test. In the case of the Creole women, only one patient had an LSIL, with negative VIA and Schiller test, but with the presence of the high-risk type 18.

## Discussion

Within the population of women included in the study, 58.3% belonged to the Eñepa ethnic group, while 41.7% belonged to the Maniapure Creole population, with an average age of 29.97 and 33.00 years, respectively. The Eñepa ethnic group showed more cervical abnormalities compared with Creole women, based on in comparison with native women, based on triple-combined testing.

There are few studies that refer to the prevalence of premalignant cervical lesions and the detection of HPV in indigenous communities, located in places that are difficult to access. However, a frequency of viral infection between 19% and 38% has been reported in indigenous communities in several countries [17]. In this study, the global frequency of HPV detection was 36.67%, with a high frequency of high-risk genotypes in individual infections. The percentage of Creole patients with a viral infection was lower than that for the indigenous population.

Nicita *et al* [18] reported the presence of HPV with a frequency of 35% in indigenous women from the municipality Alto Orinoco, Amazonas state, Venezuela, the most common genotypes being low-risk genotypes 6 and 11. On the other hand, Rodrigues *et al*, reported a frequency of HPV of 28.6% in indigenous women of Panará ethnicity, in Brazil, with 41.6% being high-risk genotypes 16, 18 or 45. They also indicate that 10.7% of the study population presented cellular atypia in the cytopathological study, reporting 22.2% for LSIL and HSIL, respectively [17]. Fonseca *et al* [14] reported a positivity rate of 34.1% for high-risk HPV 16, 31 and 18 in the Yanomami population of the Brazilian Amazon, with 5.1% of cellular abnormalities based on the cytopathological report, while Mongelos *et al* [19] indicated the presence of HPV in 16% of the Paraguayan indigenous population, with type 16 being the most common high-risk genotype, followed by type 58.

The differences observed in the frequency of detection may be due to the distribution pattern of HPV infection recorded around the world, which depends on factors such as geographical, ethnic and racial differences [20]. At the same time, they may be associated with the variation in the sensitivity and specificity of the techniques used for the detection of HPV, including the selection of primers that flank specific regions of the viral genome [21].

Regarding the age group, the patients from the indigenous group showed a statistically significant relationship between the presence of HPV in the cervix and the average age. In a cross-sectional study evaluating the prevalence of HPV in 13 countries, it was estimated that between 1.4% and 25.6% of women with normal cytology had the viral infection, with marked differences based on age range and geographical region, with a global prevalence of 10.4%. These authors conclude that HPV prevalence was higher in less developed countries compared with developed countries (15.5% versus 10.0%) and was higher in young women [22], which could explain the higher frequency of HPV in the Eñepa group, in which the average age was of 29.97 years.

Carcopino *et al* [23] indicate that 28.64% of patients under the age of 30 years were positive for the virus, with high viral loads, suggesting a relationship with recent infection, without the need for a clinical significance. In general, the curve of HPV infection is consistent in all regions of the world. This demonstrates a peak prevalence from the onset of sexual maturity to the age of 30 years, which can affect up to 80% in certain populations. This increase is due to transient infections that are quickly treated. Subsequently, it reduces and stabilizes gradually from middle age [24].

To understand these results, we must analyze them from the prevailing sociocultural point of view of these ethnic groups. The interaction between Creoles and indigenous people in these communities is evident in all aspects of the social life of the inhabitants, such as diet or biopsychosocial aspects, including sexual behaviour.

It is important to emphasize the need for health education programmes for these communities [25, 26] since we found that just over a half of the patients in this study were unaware of the existence of sexually transmitted infections. Of the women who said they were aware of them, only one mentioned HPV infection. Similarly, it is striking that only ten patients reported knowing how to avoid these STIs. Moreover, almost half of the patients stated that they were aware of contraceptive methods, which leads us to think that in these populations, in general, there is a misunderstanding in terms of differentiating between the correct use of contraceptive methods and methods for preventing STIs.

The results show the need to communicate public health policies (education, prevention and screening for STIs) to hard-to-reach communities, to overcome the cultural barriers within these communities. The combination of governmental measures, committed professionals and trained inhabitants is an important strategy to control these pathologies, developing long-term programmes that ensure adequate progress and treatment for these patients.

According to the latest US guidelines, CC screening should be carried out in all patients from the age of 21 years, with conventional or liquid phase cytology, every 3 years. Women aged 30 years and above should also be tested for high-risk HPV genotypes using molecular biology, together with cytology, and where the result is negative, the testing period can be extended to every 5 years. After the age of 65 years, screening is not indicated if the patient’s monitoring has been clear and it does not have risk factors for the development of the infection, for example, a new sexual partner [27, 28]. However, in countries such as Venezuela, Colombia and Brazil, the incidence of and mortality from CC remain high and stable, as screening is opportunistic [20].

Another important measure to take into account for effective prevention of this disease is the HPV vaccine, which is not available in the Venezuelan public system. There are three vaccines available worldwide, which protect against high-risk genotypes 16 and 18, which are principally responsible for the development of CC. Ideally, the vaccine is given to girls between 9 and 12 years of age and who are not sexually active; however, this criterion does not limit its administration. If the three doses are administered within the 6-month period as stipulated, it is reported to have up to a 99% efficacy [29].

One of the limitations of the study was the method of sample selection. It is well known that nonprobability sampling is quite common in descriptive studies [30] although it does not ensure total representation of the population as not all subjects have the same probability of being selected [31]. However, the study focused on a specific population, located in an area that is difficult to access, combined with the fact that there was only 1 month to obtain the samples, hence a nonprobability sample was proposed. Due to the beliefs and customs of the Eñepa population, the health centre was informed and the first meeting with the Chief was arranged in order to explain the objectives of the study and to obtain approval for the participation of the group of women presented. Next, the candidates were informed of the terms and design of the protocol, as well as the potential benefits, and were invited to participate.

## Conclusions

In this series, the group of indigenous women demonstrated a higher proportion of HRC, associated with cytological changes in the cervix, compared with the Creole population. Based on these findings, we recommend creating campaigns for the early detection of precancerous lesions in vulnerable and hard-to-reach populations, and which include HPV typing. This is in addition to designing programs to train health advocates who can disseminate appropriate information on the prevention of sexually transmitted infections, always respecting the customs and traditions of indigenous populations.

## Conflicts of interest

The authors have no conflicts of interest to declare.

## Figures and Tables

**Table 1.** Sociodemographic data of the Creole population and the Eñepa population included in the study.

	Creole population (*N* = 25) % (*n*)	Eñepa population (*N* = 35) % (*n*)	*P* value*
Average age	33.00 ± 11.29 years (range: 18–56)	29.97 ± 15.43 years (range: 13–67)	0.409
Marital status			0.627
Single	20% (5)	28.57% (10)	
In relationship	4% (13)	0% (0)	
Married	68% (17)	65.71% (23)	
Separated	8% (2)	0% (0)	
No answer	0% (0)	5.71% (2)	
Activity			0.683
Household work	52% (13)	45.71% (16)	
Work	28% (7)	48.57% (17)	
Student	12% (3)	0% (0)	
Unemployed	8% (2)	0% (0)	
No answer	0% (0)	5.71% (2)	
No. of couples	1.44 ± 0.58 (range: 1–3)	0.74 ± 0.44 (range: 1–2)	0.000
No. of pregnancies	4.28 ± 3.95 (range: 1–18)	2.79 ± 1.93 (range: 1–7)	0.102
Use of OC	28% (7)	2.85% (1)	0.002
Knowledge of STIs	68% (17)	5.71% (2)	0.000
Knowledge on preventing STIs	36% (9)	2.85% (1)	0.000

* *p* < 0.05

OC = oral contraceptives

**Table 2.** Screening for precancerous cervical lesions and HPV infection in the indigenous and Creole population.

	Creole population (*N* = 25) % (*n*)	Eñepa population (*N* = 35) % (*n*)	*p* value*
Schiller test	8.00% (2)	11.42% (4)	0.799
VIA	16.00% (4)	5.71% (2)	0.088
Cytology	4.00% (1)	14.29% (5)	0.999
HPV	20.00% (5)	45.71% (16)	0.031*

* *p* < 0.05

VIA = visual inspection with acetic acid, HPV = human papillomavirus
